# Application of a microbial and pathogen source tracking toolbox to identify infrastructure problems in stormwater drainage networks: a case study

**DOI:** 10.1128/spectrum.00337-24

**Published:** 2024-08-07

**Authors:** Liam R. Carson, Clint Goodman, Bert van Duin, Norman F. Neumann

**Affiliations:** 1 School of Public Health, University of Alberta, Edmonton, Alberta, Canada; 2 Community Infrastructure, City of Airdrie, Airdrie, Alberta, Canada; 3 City & Regional Planning, City of Calgary, Calgary, Alberta, Canada; Connecticut Agricultural Experiment Station, New Haven, Connecticut, USA

**Keywords:** stormwater, microbial source tracking, water pollution, fecal indicators, enteric pathogens, water quality, *Arcobacter*

## Abstract

Water scarcity and increasing urbanization are forcing municipalities to consider alternative water sources, such as stormwater, to fill in water supply gaps or address hydromodification of receiving urban streams. Mounting evidence suggests that stormwater is often contaminated with human feces, even in stormwater drainage systems separate from sanitary sewers. Pinpointing sources of human contamination in drainage networks is challenging given the diverse sources of fecal pollution that can impact these systems and the non-specificity of traditional fecal indicator bacteria (FIB) for identifying these host sources. As such, we used a toolbox approach that encompassed microbial source tracking (MST), FIB monitoring, and bacterial pathogen monitoring to investigate microbial contamination of stormwater in an urban municipality. We demonstrate that human sewage frequently contaminated stormwater (in >50% of routine samples), based on the presence of the human fecal marker HF183, and often exceeded microbial water quality criteria. *Arcobacter butzleri*, a pathogen of emerging concern, was also detected in >50% of routine samples, with 75% of these pathogen-positive samples also being positive for the human fecal marker HF183, suggesting human municipal sewage as the likely source for this pathogen. MST and FIB were used to track human fecal pollution in the drainage network to the most likely point source of contamination, for which a sewage cross-connection was identified and confirmed using tracer dyes. These results point to the ubiquitous presence of human sewage in stormwater and also provide municipalities with the tools to identify sources of anthropogenic contamination in storm drainage networks.

**IMPORTANCE:**

Water scarcity, increased urbanization, and population growth are driving municipalities worldwide to consider stormwater as an alternative water source in urban environments. However, many studies suggest that stormwater is relatively poor in terms of microbial water quality, is frequently contaminated with human sewage, and therefore could represent a potential health risk depending on the type of exposure (e.g., irrigation of community gardens). Traditional monitoring of water quality based on fecal bacteria does not provide any information about the sources of fecal pollution contaminating stormwater (i.e., animals/human feces). Herein, we present a case study that uses fecal bacterial monitoring, microbial source tracking, and bacterial pathogen analysis to identify a cross-connection that contributed to human fecal intrusion into an urban stormwater network. This microbial toolbox approach can be useful for municipalities in identifying infrastructure problems in stormwater drainage networks to reduce risks associated with water reuse.

## INTRODUCTION

It is currently estimated that by 2050, over half of the urbanized global population will be facing severe challenges posed by water scarcity due to increased urbanization, climate change, and exponential population growth (1). In addition, urbanization has led to hydromodification impacts on urban streams, which necessitates reducing the rate and volume of runoff discharged. In an effort to mitigate the strain on global water supplies and receiving water bodies, the use of alternative water sources, water recycling, and water reuse have been increasingly assessed and utilized, including the use of stormwater (2–5). However, current evidence suggests that stormwater is often of poor microbial water quality based on the levels of fecal indicator bacteria (FIB) such as *Enterococcus*, *Escherichia coli*, and total coliforms (6–18). Studies suggest that the use of traditional markers of water quality such as *Enterococcus* and *E. coli* only correlate well with gastrointestinal disease when there is an apparent point source of human sewage contamination (19–21) and not when there is no apparent point source of contamination (22–25). This can be partially attributed to the fact that FIB are non-specific to any particular animal host, being found in humans, ruminants, rodents, domestic pets, and waterfowl (26–29). Of particular concern is the public health risk posed by human sewage contamination in environmental waters, considering that the risk from this source has been generally estimated to be higher than that from other animal sources (30, 31).

Consequently, a dominant risk for stormwater use comes from enteric bacterial pathogens sourced from human and non-human feces, such as *Campylobacter* spp., *Salmonella* spp., Shiga toxin-producing *E. coli* (STEC), and *Arcobacter* (particularly *Arcobacter butzleri*), with the former three often cited as the respective top three zoonotic causes of bacterial gastrointestinal disease in humans (32, 33). At the same time, *Arcobacter* spp. (especially *Arcobacter cryaerophilus* and *A. butzleri*) have been found to be one of the most dominant pathogenic genera in human sewage (34–37).

Differentiating anthropogenic and non-anthropogenic sources of fecal pollution in stormwater becomes particularly important in characterizing the risks of human exposure to contaminated water (38, 39), and several microbial source tracking (MST) tools have been developed to investigate and help identify sources of pollution in the aquatic environment. While a number of MST technologies have been developed in recent years relying on a diversity of methods (38, 40, 41), popularity has been gaining in methods based on the use of quantitative PCR (9, 39). This method is used to detect and quantify genes or gene fragments specific to microbial populations found in particular hosts, such as the human sewage-specific HF183 marker that has been developed to effectively detect the 16S rRNA of *Bacteroides* spp. found in human feces (38, 39, 42).

Recent studies across multiple continents using MST quantitative polymerase chain reaction (qPCR) markers suggest that stormwater appears ubiquitously contaminated with human sewage, including where stormwater and sanitary sewer infrastructure are built separately (6, 9–18, 43–46). It is estimated that between 0.1% and 10% of stormwater flows may be comprised of raw human sewage based on concentrations of MST markers in stormwater and raw human sewage (13, 16, 47, 48). Moreover, a number of case studies have recently been successful in investigating and pinpointing sources of human sewage in stormwater by tracking MST markers of human sewage upstream into the drainage network until reaching a terminal point, such as at sanitary sewer infrastructure failures or illicit domestic cross-connections (6, 9, 16, 49).

Due to the limited information that can be gleaned from the enumeration of FIB alone, as well as the increasing capability of MST technologies, several jurisdictions including the province of Alberta, Canada (50), have recently implemented guidelines for stormwater use and established treatment criteria based on quantitative microbial risk assessment (QMRA) (51–53). QMRA is a bi-directional approach that can be used to estimate human health risk based on a number of factors that include an estimation of the concentration of microbial hazards often found in stormwater and can be used to set a benchmark of risk to estimate acceptable levels of microbial contamination (54). One of the most important knowledge gaps for water sources is often in the very first step of QMRA frameworks—the “hazard identification” step (54, 55). Through gathering qPCR-based estimates of human fecal sources of pollution (i.e., HF183) and enteric pathogens, the theory behind these guidelines can be more fully validated and put into practice. Recent QMRA studies have shown, for example, that even relatively low concentrations of the human fecal marker HF183 in environmental waters may increase illness risk appreciably and be detrimental to human health (56–58). In any case, few studies focusing on hazard identification through the lens of QMRA have been performed on stormwater use systems, and more work must be done to understand the sources of pollution and enteric pathogens present (2–5).

In the present study, we set out to identify fecal pollution hazards to public health in a stormwater-impacted creek (Nose Creek) in Airdrie, Alberta, Canada, and, by extension, from the use of that stormwater, by (i) assessing the presence and concentrations of common markers of human sewage contamination (i.e., HF183 and HumM2); (ii) characterizing microbial water quality in terms of FIB and comparing them to traditional recreational water quality criteria standards (59); (iii) identifying the prevalence and concentrations of select enteric bacterial pathogens in stormwater; and (iv) tracking sources of human sewage contamination in storm drainage networks to pinpoint the most likely source(s) of human fecal contamination in the drainage network.

## RESULTS

### Microbial water quality based on fecal indicators

**Fig 1:**
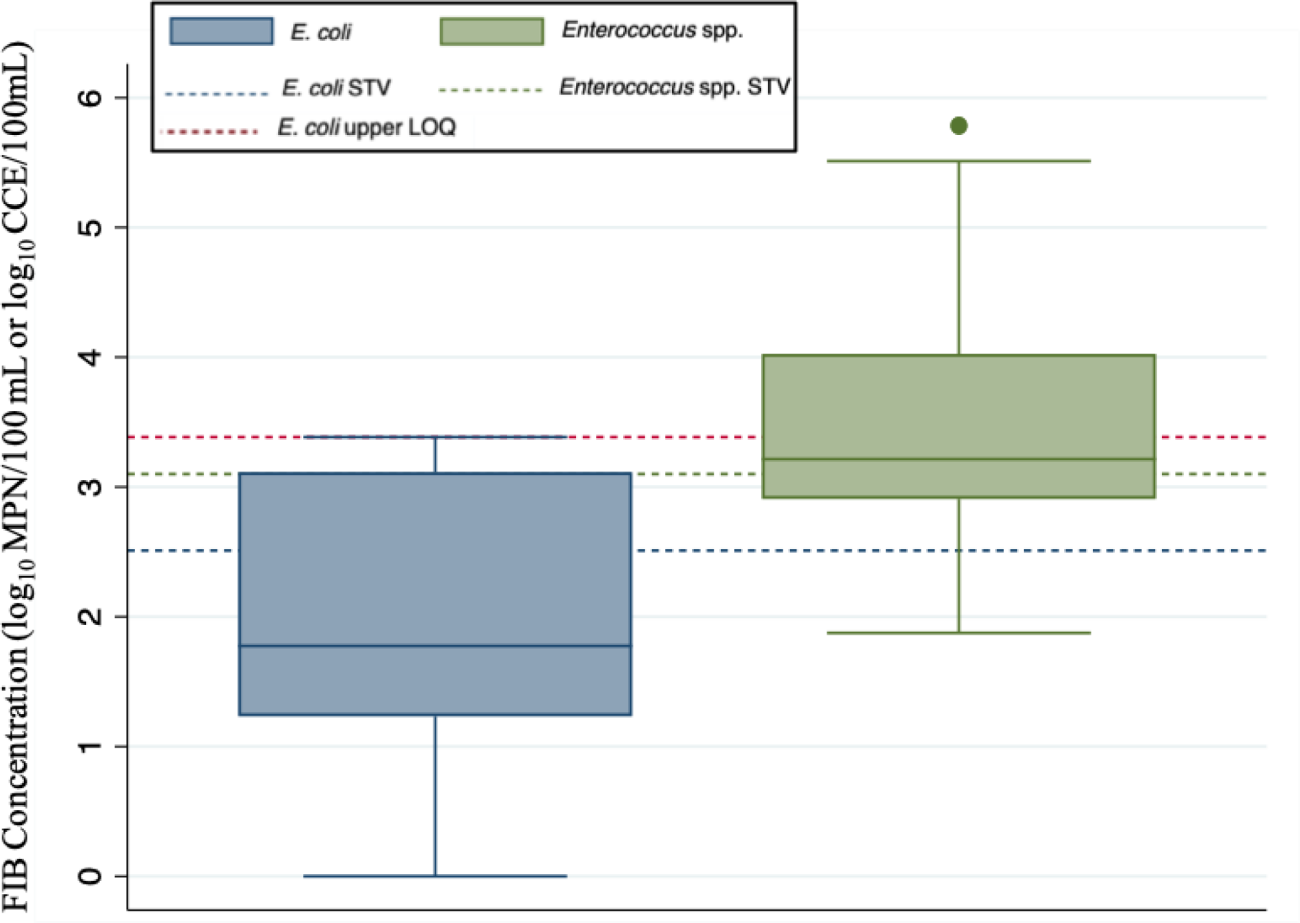
Box and whisker plots of total FIB distributions in all sampled routine stormwater outfalls combined from Nose Creek in Airdrie, Alberta, Canada (sampled in 2021). The *E. coli* distribution is represented in blue and the *Enterococcus* distribution in green. The solid line within each box is representative of the median FIB concentration, and the upper and lower horizontal edges of each box represent the 25th and 75th percentile values of concentration, while whiskers represent ±1.5*interquartile range. Outliers are represented by colored dots outside the range of the upper whisker. Note the dotted lines (blue for *E. coli*, green for *Enterococcus*) representing acceptable recreational water quality criteria for *E. coli* (320 MPN/100 mL) and *Enterococcus* (1,280 CCE/100 mL) (59), as well as the red dotted line representing the upper limit of quantification of the Colilert assay (2,419.60 MPN/100 mL).

Not surprisingly, all FIB (*E. coli*, *Enterococcus*, and total coliforms) were found in 100% of samples and at relatively high concentrations from all storm outfall samples in Nose Creek (though especially *Enterococcus*—see Fig. 1). For example, total coliforms were frequently found to be ≥3.4 log_10_ most probable number (MPN)/100 mL (the upper limit of the assay) and in 32 of 38 (84.2%) of routine samples. *Enterococcus* and *E. coli* had respective geometric means of 3.5 log_10_ cell calibrator equivalent (CCE)/100 mL (range: 1.9 log_10_ CCE/100 mL–5.8 log_10_ CCE/100 mL) and 2.0 log_10_ MPN/100 mL (range: <1 log_10_ MPN/100 mL–>3.4 log_10_ MPN/100 mL). Despite differences in magnitude, these FIB were found to be highly correlated to each other in routine outfall samples based on the Spearman rank test (see Table 1). Nose Creek sites frequently exceeded traditional water quality guidelines, such as those set by the U.S. EPA for recreational water quality (59). The majority of samples (21 of 38 samples, 55.3%) exceeded the *Enterococcus* statistical threshold value (STV) of 1,280 CCE/100 mL, while the site-specific *Enterococcus* geometric mean (GM) of 300 CCE/100 mL was exceeded at every single site (see Table 2). *Escherichia coli* criteria exceedance was less frequent than for *Enterococcus* at the routine Nose Creek sites, though every site except NP#1 had at least one sample exceed the *E. coli* STV of 320 MPN/100 mL, and six of nine (66.6%) outfalls studied also exceeded the site-specific *E. coli* GM of 100 MPN/100 mL. Importantly, FIB criteria exceedance did not always converge with human sewage detection, with *E. coli* concentrations exceeding STV criteria in only 7 of 22 samples (31.8%) that were also positive for HF183, while *Enterococcus* concentrations were above their respective STV in 11 of 22 (50.0%) samples positive for HF183.

**TABLE 1:** Spearman correlation coefficients for FIB routinely sampled from Nose Creek stormwater outfalls in Airdrie, Alberta (*n* = 38)

Correlation (ρ)		
FIB	Total coliforms	*E. coli*
*Enterococcus* spp.	0.52^*b*^	0.81^*a*^
*E. coli*	0.65^*a*^	N/A

^*a*^ *P* < 0.0001.

^*b*^ *P* < 0.001.

**TABLE 2:** Occurrences of human sewage marker HF183 and *A. butzleri* marker (*hsp60*) alongside U.S. EPA (59) recreational water quality criteria exceedances (boldface representing criteria that were violated) for routine stormwater outfall samples taken at Nose Creek outfalls (*n* = 38) in Airdrie, Alberta, Canada

Nose Creek site	*n*	*A. butzleri* marker (*hsp60*) frequency (%)	HF183 marker frequency (%)	*A. butzleri* and HF183 marker co-detection frequency (%)	*Enterococcus* site GM (log_10_)^*a*^	*E. coli* site GM (log_10_)^*a*^	*Enterococcus* site STV exceedance (%)^*b*^	*E. coli* site STV exceedance (%)^*b*^
N#1	5	1/5 (20.0)	5/5 (100.0)	1/5 (20.0)	**3.29**	**2.03**	**2/5** (**40.0**)	**2/5** (**40.0**)
N#2	4	4/4 (100.0)	3/4 (75.0)	3/4 (75.0)	**3.59**	**2.18**	**3/4** (**75.0**)	**1/4** (**25.0**)
N#3	4	2/4 (50.0)	1/4 (25.0)	1/4 (25.0)	**3.44**	**2.18**	**2/4** (**50.0**)	**1/4** (**25.0**)
N#4	4	3/4 (75.0)	3/4 (75.0)	3/4 (75.0)	**4.19**	**2.81**	**3/4** (**75.0**)	**2/4** (**50.0**)
N#5	4	2/4 (50.0)	3/4 (75.0)	2/4 (50.0)	**3.73**	**2.48**	**2/4** (**50.0**)	**2/4** (**50.0**)
N#6	4	3/4 (75.0)	2/4 (50.0)	2/4 (50.0)	**3.85**	**2.54**	**4/4** (**100.0**)	**2/4** (**50.0**)
N#7	4	2/4 (50.0)	1/4 (25.0)	1/4 (25.0)	**2.87**	1.53	**1/4** (**25.0**)	**1/4** (**25.0**)
N#8	4	3/4 (75.0)	3/4 (75.0)	3/4 (75.0)	**3.49**	1.83	**2/4** (**50.0**)	**1/4** (**25.0**)
NP#1	5	1/5 (20.0)	1/5 (20.0)	0	**3.08**	1.07	**2/5** (**40.0**)	0
Total	38	21/38 (55.3)	22/38 (57.9)	16/38 (42.1)	N/A	N/A	21/38 (55.3)	12/38 (31.6)

^*a*^ *Enterococcus* and *E. coli* GM of 300 CCE/100 mL and 100 MPN/100 mL, respectively, were used as water quality criteria, with bold values indicating those that exceeded these criteria (59).

^*b*^ *Enterococcus* and *E. coli* STVs were 1,280 CCE/100 mL and 320 MPN/100 mL, respectively, with bold values indicating those sites where >10% of samples exceeded these values (59).

### Evidence of human sources of fecal pollution impacting stormwater

**Fig 2:**
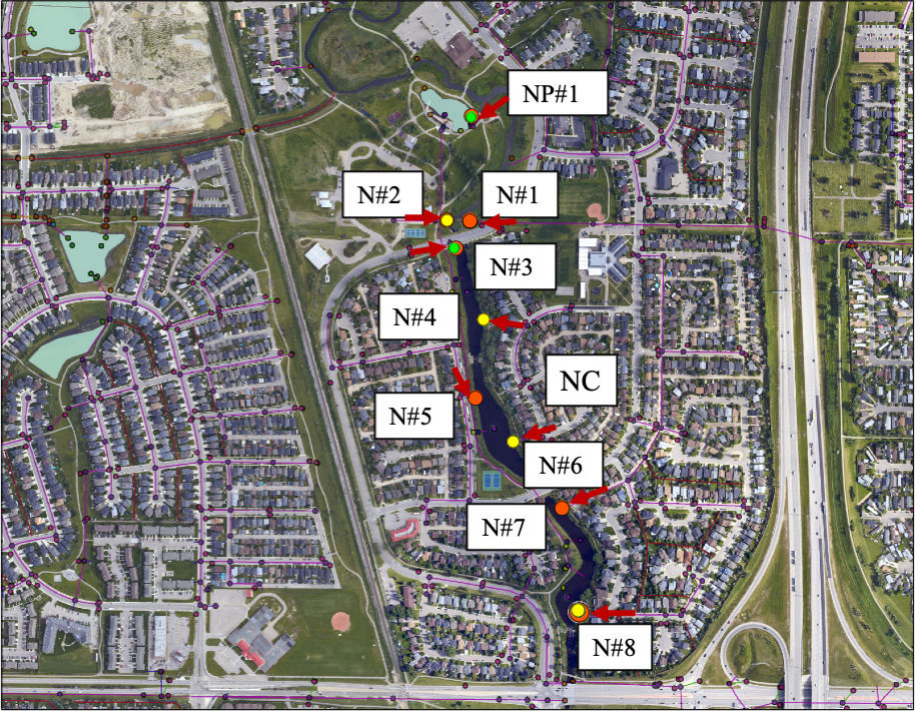
Map of human sewage marker (HF183) detection within stormwater outfalls into Nose Creek (NC) in Airdrie, Alberta, 2021. Note the overlaid colored dots representative of HF183 detection at quantifiable levels (red), detectable but not quantifiable (yellow), and not detected (green) in the Nose Creek Pond (NP#1) and Nose Creek sites (NC#1–8). Red arrows represent the directional flow of stormwater. Note that all maps presented were created and co-owned with the City of Airdrie, and have been used with permission.

Human fecal contamination of stormwater, as determined by the presence of the human fecal marker HF183, was detected at every stormwater outfall at least once and, overall, was detected in the majority of routine samples taken from Nose Creek in 2021 (22 of 38 samples or 57.9%) (Fig. 2; Table 2). However, most samples positive for HF183 in Nose Creek were found to have very low concentrations of the marker [detectable but non-quantifiable (DNQ) in 13 of 22 samples (59.1%)], though this marker ranged from 3.6 log_10_ copies/100 mL to 4.3 log_10_ copies/100 mL when detected at quantifiable levels. In contrast, the other human fecal marker used (HumM2) was only detected in 6 of 38 (15.8%) total routine samples from Nose Creek and always at a DNQ concentration. While the majority of samples positive for HumM2 were also positive for HF183 (6 of 8 samples, 75.0%), only 27.3% of total samples positive for HF183 (6 of 22 samples) were also positive for HumM2. The higher concentrations found for HF183 compared to HumM2 suggested that HF183 was potentially a more sensitive indicator of human sewage than HumM2. Regardless, the presence of both markers suggested that human fecal wastes were frequently, albeit sporadically, flowing into Nose Creek from stormwater.

### Enteric bacterial pathogens

Given that (i) human feces was identified as an important source of microbial pollution flowing into Nose Creek and (ii) pathogens such as *Arcobacter* spp. are abundant in municipal sewage, we sought to better understand risk by evaluating bacterial pathogen levels in stormwater. *Arcobacter butzleri* and *Campylobacter* spp. were detected in 55.3% (21 of 38) and 7.9% (3 of 38) of routine samples collected at Nose Creek outfalls, respectively. *Arcobacter butzleri* was detected at least once at all Nose Creek outfalls studied. In the 21 samples positive for *A. butzleri*, six samples had reasonably high quantifiable concentrations, ranging from 3.9 to 4.1 log_10_ copies/100 mL. In contrast, all three samples positive for *Campylobacter* spp. were at low levels (i.e., DNQ). *Salmonella* and STEC were not detected in stormwater flowing into Nose Creek at any of the outfall sites tested.

Of the 21 samples positive for *A. butzleri*, 16 samples (76%) were also positive for human fecal contamination based on HF183 (see Table 3). An independent analysis of these 16 samples across the 38 (42.1%) total number of stormwater effluent samples flowing into Nose Creek revealed that *A. butzleri* and HF183 were statistically significantly more likely to be detected together than for either marker to be detected alone [based on Fisher’s exact test (*P* = 0.013)]. This was in contrast to *Campylobacter*, where none of the positive samples were also positive for HF183. *Enterococcus* STV criteria exceedance occurred in 14 of 21 (66.7%) samples positive for *A. butzleri*, whereas the *E. coli* STV criteria were only exceeded in 8 of 21 (38.1%) routine outfall samples positive for this pathogen.

**TABLE 3:** Two-by-two table of Nose Creek 2021 routine Airdrie stormwater samples positive for *A. butzleri* (*hsp60*), human sewage marker (HF183), both, or neither (*n* = 38)

	HF183 detected	HF183 not detected
*A. butzleri* detected	16	5
*A. butzleri* not detected	6	11

### Investigating point sources of human fecal pollution in the stormwater drainage network

**Fig 3:**
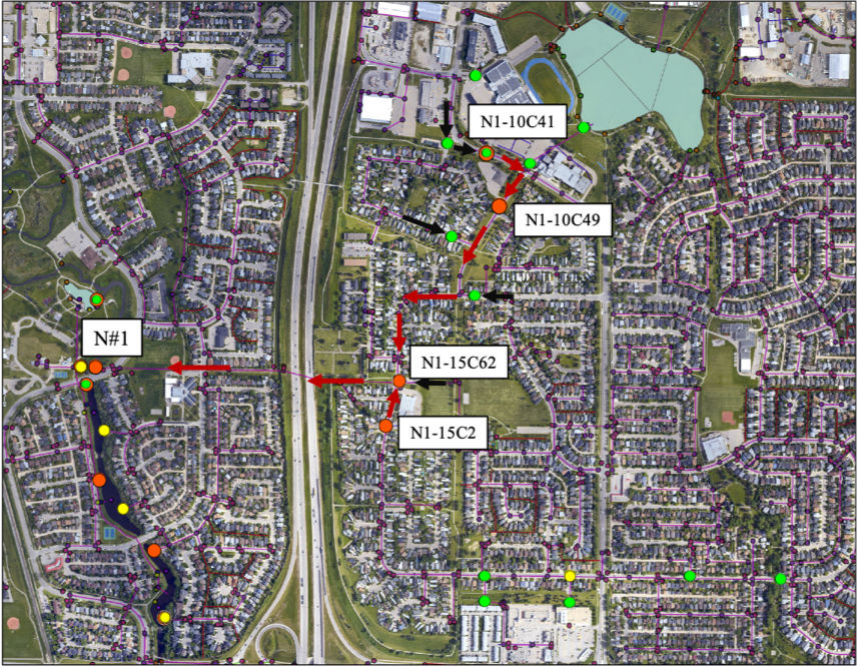
Map of Nose Creek stormwater drainage network summarizing the most relevant manholes tested for the human sewage marker HF183 upstream of the positive outfall site labeled N#1. Note the manholes upstream of N#1 where HF183 was positive and quantifiable (red dots), detectable but not quantifiable (yellow dots), or not detected (green dots). Also note the directional flow of stormwater through the system, represented by drainage networks positive for HF183 (red arrows) as well as stormwater flows with no demonstrable HF183 detection (black arrows). Note that all maps presented were created and co-owned with the City of Airdrie, and have been used with permission.

Stormwater effluent at the most upstream site on Nose Creek (i.e., Site N#1—see Table 2; Fig. 2) was consistently contaminated with human sewage based on the presence of the human fecal marker HF183. Subsequently, several manholes were systematically sampled upstream of the N#1 stormwater outfall, for which we observed a distinct pattern in both the presence of HF183 and an increasing concentration gradient toward the more northern and distant manholes sampled during the investigation (e.g., Manhole N1-10C41 in the northeast quadrant of the city) (Fig. 3). A manhole (N1-15C62) immediately upstream of the outfall N#1 had approximately a 10-fold increase in HF183 concentration compared to the stormwater effluent flowing into the creek at N#1 itself (i.e., 4.4 log_10_ copies/100 mL versus 3.6 log_10_ copies/100 mL, respectively; Fig. 3; Table 4) and which appeared to come from the north trunk of the storm drain feeding into this manhole. Testing water quality in manholes upstream of N1-15C62 revealed an increasing concentration of HF183 in the north trunk sewer, peaking at 5.4 log_10_ copies/100 mL at Manhole N1-10C49 and 6.1 log_10_ copies/100 mL at Manhole N1-10C41 (Fig. 3; Table 4). Interestingly, HumM2 (generally a less sensitive human marker of fecal contamination compared to HF183), was also detected at these two upstream manholes at relatively large concentrations (i.e., 4.3 log_10_ copies/100 mL at Manhole N1-10C49 and 6.1 log_10_ copies/100 mL at Manhole N1-10C41), which was not detected at downstream manholes nor the outfall into Nose Creek. This “closing-in” on the point of contamination using MST markers was reinforced by FIB concentrations, whereby levels of *E. coli* at N1-10C49 and N1-10C41 exceeded the upper limits of detection by the Colilert Quanti-Tray method but did not exceed this limit at the downstream Manhole N1-15C62 or the outfall flowing into Nose Creek (Table 4). Likewise, increasing concentrations of *Enterococcus* were also observed peaking at Manhole N1-10C41 [compare *Enterococcus* levels at N#1 in Nose Creek (2.2 log_10_ CCE/100 mL) to Manhole N1-10C41 (5.6 log_10_ CCE/100 mL)]. Sampling manholes further upstream of Manholes N1-10C49 and N1-1041 revealed no evidence of human fecal pollution and lower FIB counts coming from other directions, suggesting that the actual physical source of contamination was in the vicinity of Manhole N1-10C41. As a result, the City of Airdrie conducted a fluorescein tracer dye test on a nearby multi-user recreational building and confirmed that certain toilets within this facility had been plumbed into the stormwater drains feeding into Manhole N1-10C41. Consequently, this was deemed to be the primary source of human fecal contamination draining into Nose Creek.

**TABLE 4:** MST and FIB results from select investigative samples highlighting the most direct proposed path of human fecal contamination from a local community center in Airdrie, Alberta, to the N#1 outfall feeding Nose Creek

Sampling date (date/month/year)	Site	HF183	HumM2	*Enterococcus*	*E. coli*
		log_10_ copies/100 mL	log_10_ copies/100 mL	log_10_ CCE/100 mL	log_10_ MPN/100 mL
7/9/21	N#1	3.64	ND	2.19	0.98
8/9/21	N1-15C62-N	4.40	ND	2.23	1.30
27/9/21	N1-10C49-S	5.36	4.31	4.36	>3.38^*a*^
	N1-10C41-N	6.08	6.05	5.60	>3.38^*a*^

^*a*^ Greater than the upper limit of detection (LOD) of assay.

Our investigations also suggested that the southern trunk of the drainage network flowing into Manhole N1-15C62 (i.e., the manhole immediately upstream of N#1 site draining into Nose Creek) also contributed human sewage into Nose Creek, albeit far less than that coming from the north trunk (see Table S1; Fig. S1). Samples taken the same day at Manhole N1-15C62 and an upstream manhole immediately south (i.e., Manhole N1-15C2) revealed that both these manholes contained HF183, but concentrations were far greater at N1-15C62 (5.3 log_10_ copies/100 mL) compared to N1-15C2 (4.1 log_10_ copies/100 mL). This result was also reflected in the higher *E. coli* concentrations seen at N1-15C62 compared to N1-15C2. Likewise, HumM2 was detected at N1-15C62 at a relatively high concentration (4.32 log_10_ copies/100 mL), but only at detectable but non-quantifiable levels at N1-15C2 (Table S1). Testing of manholes further to the south resulted in several non-detects, and only one detectable but non-quantifiable sample for HF183 (Table S2). Collectively, the data suggested that the bulk of the human fecal contamination entering Nose Creek through outfall N#1 was due to the cross-connection in the multi-user recreational facility in the north part of the city but that minor contributions of human fecal contamination were coming from the south as well (albeit sources remain unknown).

## DISCUSSION

Stormwater is generally considered to be a relatively poor-quality water source, particularly for municipalities that manage stormwater using combined sewer outfalls (CSOs) (60). Nevertheless, even in municipalities where stormwater and sanitary sewers are completely separate, human sewage contamination can be fairly widespread, albeit often at sporadic and frequently low levels. Sources of human fecal pollution within these separated systems can be diverse, ranging from infrastructure failures (e.g., leaky sewer systems or service connections and inadvertent cross-connections), illicit discharges (e.g., dumping of recreational vehicle wastes), or even unhoused populations within urban municipalities.

The City of Airdrie in Alberta, Canada, represented an ideal case study for characterizing sources of urban pollution in a fully separated stormwater drainage system. Herein, we demonstrate that for stormwater outfalls persistently contaminated with human fecal markers, MST, FIB, and pathogen-based monitoring tools can be useful for pinpointing the physical source of pollution within the storm drainage network. In particular, we identified a consistent yet low-level signature of the human fecal marker HF183 (~10^3^ copies/100 mL) flowing from stormwater effluents (both during base flow and storm flows) into an urban creek and tracked these molecular signatures upstream through the drainage network. In some effluent samples, the levels of *E. coli* and *Enterococcus* were low and met recreational water quality criteria, yet they still contained a persistent HF183 signature, indicating a potential structural problem in the upstream drainage network that might pose a risk to human health. We subsequently monitored the stormwater quality in the drainage network upstream of this “contaminated” outfall, which revealed an ever-increasing concentration in HF183 as we moved further upstream within the drainage network. HF183 levels peaked at >10^6^ copies/100 mL—a concentration approximately 1,000× greater than that observed in the effluents flowing into the creek, which also correlated with an increase in FIB concentrations (i.e., >250× and >2,500× increase in *E. coli* and *Enterococcus* concentrations, respectively). At the most contaminated site in the drainage network, the human fecal marker HumM2 was also detected, even though it was not detected in the effluents flowing into the urban creek. HumM2 has been reported to be less sensitive than HF183, due to the HumM2 marker not always being detected within individual human fecal samples (61), and often typically at lower concentrations (by at least one to two orders of magnitude) in human feces and sewage in comparison to HF183 (62, 63). Collectively, this suggested that the contamination source impacting the storm drainage network was in the near vicinity, and tracer dye testing at a nearby multi-user recreational facility confirmed that several toilets within the facility were cross-connected into the storm drains. This toolbox approach to microbial water quality investigations proved to be extremely valuable in pinpointing infrastructure problems in municipal storm drains that are fully separated from municipal sewage, and offers a novel approach for municipalities to manage cross-connection programs and monitor for infrastructure problems in storm drainage networks.

The prevalence of HF183 in stormwater in Nose Creek is consistent with what is found in other studies around the world, though there is also high variability in marker concentrations (<2 log_10_–>6 log_10_ copies/100 mL) (4, 6, 9–11, 13, 16–18, 44, 46). This highlights the variability of this marker in stormwater and reflects the different sources of fecal pollution that range from low-impact cross-connections at single-family residential households to broken sanitary sewer pipes (6, 44, 45). As concentrations of HF183 are typically 7–8 log_10_ copies/100 mL in raw human municipal sewage (13, 16, 47, 48), our data suggest that approximately 0.1% of the total stormwater effluent (i.e., 1 of every 1,000 L) flowing into the urban creek at this site was made up of raw human sewage.

Recently, multiple similar studies have used the MST toolbox approach to investigate stormwater drainage networks upstream of contaminated outflows, finding similar results [see references (6, 9, 16, 49)]. Three case studies by Gonzalez et al. (49) and one by Hachad et al. (9) were able to pinpoint and identify specific areas of human sewage contamination leakage that included sanitary sewer infrastructure failures, sanitary sewer blockages, and illicit cross-connections. Along with the current case study at Nose Creek, this highlights the effective use of MST technology by municipalities to mitigate fecal pollution and consequent public health risks of stormwater use.

Overall, the microbial water quality of Airdrie stormwater flowing into Nose Creek was found to be generally poor based on traditional (FIB) water quality guidelines/standards, and this is consistent with the literature, which demonstrates the persistent and near-universal exceedance of recreational or ambient water quality FIB criteria in stormwater (6–18). The correlation between *Enterococcus* and *E. coli* FIB was significant and relatively high (ρ ≥ 0.8) in Nose Creek outfall samples, being similar to several studies elsewhere (15, 17, 44), though other publications found only weak to moderate correlation between these two (6, 11, 64).

It is notable that high rates of FIB criteria exceedance did not necessarily always co-occur in samples where human sewage marker HF183 was consistently detected, and several studies report relatively low to moderate correlation between the two (9, 13, 14, 18, 45, 46), while Sauer et al. (16) found no significant correlation at all. Differing decay rates between FIB and HF183 (47, 65, 66) as well as animal-specific sources of FIB [see references (26, 27)] may also be contributing to fecal pollution in the drainage network, and indeed, in our study, multiple sources of fecal pollution could be observed (Carson LR, Beaudry M, Valeo C, He J, Banting G, van Duin B, Goodman C, Scott C, and Neumann NF, submitted for publication). These data suggest that FIB monitoring alone is insufficient as a tool for identifying and pinpointing possible infrastructure problems in storm drainage systems and that these methods should be combined with other tools, such as MST (i.e., using human sewage marker HF183 where human sewage contamination is a concern).

While most enteric pathogens were not (or were rarely) observed in the current study (including *Campylobacter*, *Salmonella*, and STEC), *A. butzleri* was observed frequently in Nose Creek samples, found in 21 of 38 samples, and in 16 of these *A. butzleri* positive samples, HF183 was also detected, reinforcing the finding that human sewage is likely a major source of this pathogen. *Arcobacter* spp. are abundant (and dominant) in human sewage (35–37) and have been found to correlate well with markers of human sewage in receiving water bodies heavily contaminated by human fecal pollution (34, 67). In some cases, *Arcobacter* spp. were also found to correlate relatively well with FIB in sewage-contaminated environmental waters (68, 69). This is consistent with the present study, where *A. butzleri* appeared to be a dominant pathogen in stormwater impacted by human sewage. By contrast, other enteric bacterial pathogens, such as *Campylobacter* spp., STEC, and *Salmonella* spp., are often only sporadically detected in stormwater and at relatively low concentrations (43, 70) and are often found to correlate poorly to FIB (8, 64, 70) and human sewage markers (34, 45, 64). These pathogens are often used in QMRA modeling studies as reference pathogens for evaluating risk, but our data, however, suggest that *A. butzleri* may be far more abundant in stormwater than these commonly used reference pathogens, warranting some consideration for *Arcobacter* to be used as a potential reference pathogen for QMRA studies on stormwater use. Unfortunately, few studies have looked into the presence of pathogenic *Arcobacter* spp., such as *A. butzleri*, in stormwater (67, 71). In the study by Beaudry (71), approximately 75% of stormwater samples analyzed contained culturable *Arcobacter butzleri*, and based on virulence gene analysis, these isolates appeared to represent pathogenic strains of the bacteria.

As stormwater use and water reuse systems have not yet been extensively studied in terms of risk (2–5), understanding the microbial hazards inherent within these systems is of paramount importance. For example, according to several QMRA studies, concentrations of HF183 hovering around 3–4 log_10_ copies/100 mL in the stormwater ponds themselves (as opposed to in upstream outfalls, where concentrations may be even higher) can be hazardous to human health (56–58), highlighting the increased need to better understand potential human sewage levels in stormwater systems designated for use even when FIB levels do not exceed guideline/regulatory criteria. Schoen et al. (5) also found that risks of gastrointestinal illness could vary for different non-potable uses of stormwater, further suggesting the complex number of factors to consider when exposing people to stormwater as an alternative water source.

In conclusion, the current study improves our understanding of the microbial hazards present in stormwater and promotes the use of a microbial toolbox approach to monitoring FIB, MST, and pathogen occurrence for identifying and mitigating environmental contamination risks in the urban water environment.

### Conclusions and recommendations

- Stormwater can be a low-quality water source and may not meet ambient or recreational water quality criteria (i.e., *Enterococcus* and *E. coli* counts).
- Human sewage contamination (as measured by HF183) can be commonly observed in stormwater, even in systems separated from sanitary.
- MST, FIB, and pathogen-related methods can be successfully employed to pinpoint the source of human fecal pollution in a storm drainage network.
- The bacterial enteric pathogen *A. butzleri* (but not *Campylobacter* spp., STEC, or *Salmonella* spp.) was commonly found in stormwater and significantly associated with human markers of fecal pollution.

## MATERIALS AND METHODS

### Sampling area and strategy

Two sampling strategies were used when testing stormwater from Nose Creek in the City of Airdrie, Alberta, which consisted of (i) routine outfall sampling (bi-weekly) and (ii) investigative sampling where we interrogated the urban stormwater drainage network (i.e., from downstream outfalls to manholes further upstream) to pinpoint the physical source(s) of sewage contamination when human MST markers of pollution (HF183 and HumM2) were observed. The routine stormwater sampling sites consisted of eight separate outfalls (labeled N#1–N#8) draining into Nose Creek within the city limits, as well as one outfall draining into the Nose Creek Pond (NP#1), which is attached to the creek (see Fig. 2). Each site was sampled on four separate dates (with the exception of N#1 and NP#1, which were sampled on five occasions), for a total of 38 routine samples collected over 4/5 weeks on an approximately bi-weekly basis from 20 July to 7 September 2021.

Investigative sampling of manholes upstream of those outfalls (i.e., N#1) found to be contaminated with human sewage was also performed, with a total of 30 individual samples collected from 15 manholes upstream of this site. Investigative sampling was done between 26 July and 27 September 2021, on an approximately bi-weekly basis. During the investigation, samples were taken when the (base) flow was sufficient and from multiple trunks running in different directions upstream.

Sample collection for both routine and investigative samples consisted of 200 mL grab samples collected in sterile bottles either directly from the stormwater outfall (routine sampling) or directly from pipes flowing into stormwater manholes (investigative sampling). Samples were then shipped overnight on ice from Airdrie to the University of Alberta, in Edmonton, where the samples were fully processed within 24 hours of being collected.

### Microbial culture methods

*Escherichia coli* and total coliforms were enumerated by the defined substrate methods using Colilert in a Quanti-Tray MPN format (Idexx Laboratories, Inc.; Westbrook, ME, USA), as per the manufacturer’s instructions. Briefly, 100 mL of stormwater sample was added to a small vessel alongside one packet of Colilert reagent, then inoculated into a Quanti-Tray/2000 system and incubated at 35°C for 24 hours. Positive and negative controls, respectively, consisted of 100 mL of sterile deionized water that underwent the same process as above but was either spiked with one colony of *E. coli* ATCC 25922 [incubated previously for 24 hours on trypticase soy agar (BD; Thermo Fisher Scientific, Ottawa, Ontario, Canada) at 37°C for 24 hours] or was not spiked and left as sterile water only. After this 24-hour incubation, results were calculated using a standard MPN table based on a yellow-color change within Quanti-Tray cells (total coliforms) and additional fluorescence under long-wave UV (*E. coli*).

### Molecular-based detection and quantification methods

Quantitative PCR methods based on TaqMan chemistry were used to test for several markers, including for human sewage [HF183 and HumM2—see references (42, 72)], the FIB *Enterococcus* (73, 74)*,* and enteric pathogens including *A. butzleri* (75), *Campylobacter* spp. (76), *Salmonella* spp. (77), and STEC (78) (also see Table S3).

### Sample preparation for qPCR

Stormwater samples were prepared for qPCR testing by first filtering 20 mL of each sample through disposable 0.4-µm pore polycarbonate MicroFunnel filters (Pall Corporation, New York, USA). Sample filters were then extracted and processed according to U.S. EPA Method 1611 (74). Briefly, sample filters [including three filtered calibrators and one filtering blank per day of sampling as described elsewhere (74)] were added to bead tubes (Generite, North Brunswick, NJ, USA) alongside AE buffer (10 mM Tris-Cl, 0.5 mM EDTA, pH 9.0) (Qiagen; Hilden, Germany) and 0.2 µg/mL of *Oncorhynchus keta* (salmon) sperm as an internal control (74, 79). Tubes were homogenized by a Bead Mill 24 Homogenizer (Thermo Fisher Scientific, Waltham, MA, USA), and the resulting supernatant was transferred to separate tubes and centrifuged twice before the final supernatant was used as the DNA template for qPCR. Templates were frozen at −80°C until qPCR testing.

### qPCR reaction conditions

An Applied Biosystems 7500 Real-Time PCR (Applied Biosystems; Thermo Fisher Scientific, Ottawa, Ontario, Canada) was used for the performance of all qPCR assays and under fast cycling conditions. All qPCR runs consisted of two-step reactions with the following cycling conditions: 3 minutes of initial denaturation at 95°C, before denaturation and annealing/extension, respectively, at 95°C for 5 seconds and 60°C for 30 seconds for 40 cycles. Reagents used for all runs included 1× PrimeTime Gene Expression Master Mix (Integrated DNA Technologies, Coralville, IA, USA), 200 µg/mL bovine serum albumin (Sigma-Aldrich, St. Louis, MI, USA), and the appropriate primers and probe concentrations dependent on the assay (see Table S3). Primers and probes for all markers are described in Table S3. All reactions consisted of 15 µL of the above reagents and 5 µL of DNA template/control for a total of 20 µL per reaction. All assays were set at a fluorescence threshold of 0.1 with the exception of the VD16S (*Campylobacter* spp.) marker, which was run at a threshold of 0.05. MicroAmp Fast Optical 96-well plates (Applied Biosystems, Foster City, CA, USA) were used for all qPCR runs; samples and negative controls (no-template controls, filtering blanks) were run in duplicate wells, while positive controls (calibrators or plasmids as appropriate) were run in triplicate wells.

### qPCR controls

Positive controls for qPCR reactions consisted either of calibrators or a series of plasmid standard dilutions, depending on whether *Enterococcus* or qPCR markers for human feces/enteric pathogens were being enumerated, respectively. More specifically, calibrators were used when measuring sample inhibition or determining the concentrations of *Enterococcus* via the ΔΔCT relative quantification method as specified in U.S. EPA Method 1611 (74). Appropriate plasmid standards were instead used in dilutions of 50K copies/5 µL to 5 copies/5 µL for absolute quantification of other markers, including two human sewage markers (HF183 and HumM2) as well as enteric pathogen markers including those for *A. butzleri*, *Campylobacter* spp., *Salmonella* spp., and STEC (see Table S3). Negative controls consisted of filtering blanks (i.e., filtered sterile phosphate-buffered saline) and no-template controls (i.e., nuclease-free water), while salmon sperm (*O. keta*) was used as an inhibition control (74, 79). Samples displaying a shift of >3 Cts of salmon sperm concentration in comparison to calibrators were considered inhibited, as specified in U.S. EPA Method 1611 (74), and diluted with water to 1:5 and 1:25 dilutions before being run again for qPCR analysis.

### Data analysis

Prior to data analysis, estimates of qPCR markers (both MST and enteric pathogens), *Enterococcus*, and *E. coli* were all reported, respectively, as either copy, CCE, and MPN per 100 mL of stormwater sampled. Data analysis began with first log_10_ transforming quantifiable estimates of FIB, MST markers, and enteric pathogen markers. As some samples for *E. coli* were at the lower (<1 MPN/100 mL) or upper (>3.4 log_10_ MPN/100 mL) detection limits of the Colilert assay, these samples were simply set to these limits for the sake of analysis. In terms of qPCR testing, marker estimates were considered either non-detects (ND) if the marker amplification was absent over 40 cycles, DNQ if amplification occurred but marker estimates were found to be below the 95% percentile of the limit of detection (LOD_95_), or quantifiable if marker estimates were found to exceed this limit. Testing by the Shapiro–Wilk test found that FIB were not normally distributed. As a result, the non-parametric Spearman rank test was used to determine if there was any significant correlation between *Enterococcus*, *E. coli*, and/or total coliforms, while the non-parametric Fisher’s exact test was used to determine whether HF183 and *A. butzleri* were significantly more likely to be detected within the same sample, rather than independently.

## SUPPLEMENTAL MATERIAL

Supplemental material spectrum.00337-24-s0001.pdf Fig. S1; Tables S1 to S3.

ASM does not own the copyrights to Supplemental Material that may be linked to, or accessed through, an article. The authors have granted ASM a non-exclusive, world-wide license to publish the Supplemental Material files. Please contact the corresponding author directly for reuse.
