# Supplementary material for: Application of a microbial and pathogen source tracking toolbox to identify infrastructure problems in stormwater drainage networks: a case study
Source: Microbiol Spectr. 2024 Aug 7;12(9):e00337-24. doi: 10.1128/spectrum.00337-24 (PMC11371268; doi:10.1128/spectrum.00337-24)
Supplement: Supplemental material — Fig. S1; Tables S1 to S3. [file spectrum.00337-24-s0001.pdf]

SUPPLEMENTAL MATERERIAL:

Application of a Microbial and Pathogen Source Tracking 'Toolbox' to Identify Infrastructure Problems in Stormwater Drainage Networks: A Case Study

Liam R. Carson [a], Clint Goodman [b], Bert van Duin [c], & Norman F. Neumann [a#]

[a] School of Public Health, University of Alberta, Edmonton, Alberta, Canada

[b] Community Infrastructure, City of Airdrie, Airdrie, Canada

[c] City & Regional Planning, City of Calgary, Calgary, Alberta, Canada

# Corresponding Author: Dr. Norman F. Neumann, PhD
Professor
Room 3-57E, South Academic Building,
School of Public Health
University of Alberta,
Edmonton, Alberta, Canada.
T6G 2E9
Email: nfneuman@ualberta.ca

Supplemental Information:

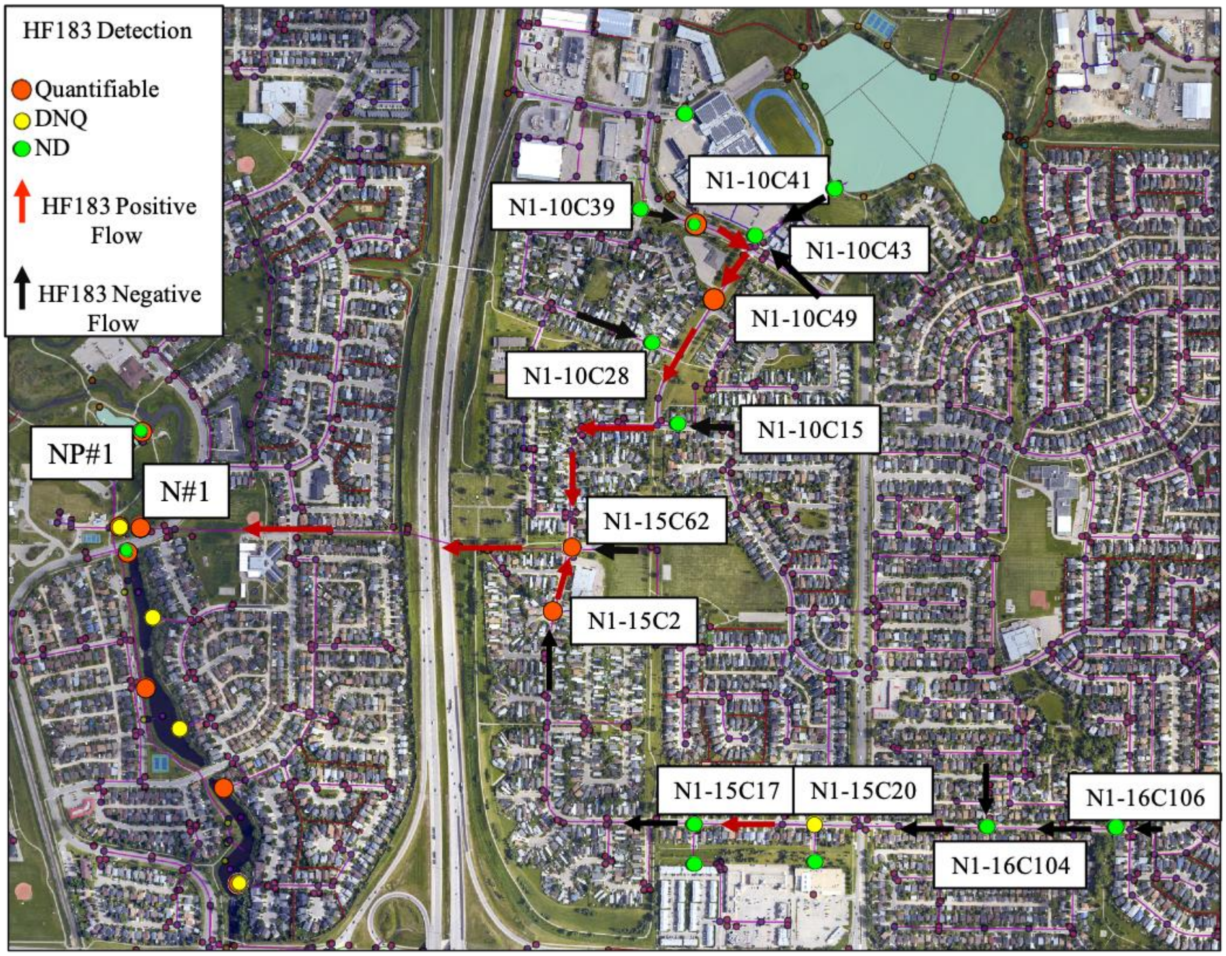

Fig. S1 Map of Nose Creek stormwater drainage network and the manholes tested for human sewage marker HF183 upstream of HF183 positive outfall site N#1 from the final two weeks of the investigation. Note the manholes upstream of N#1 where HF183 was positive and quantifiable (red dots), detectable but not quantifiable (yellow dots), or not detected (green dots). Note also the flow of stormwater through the system, represented by presumed HF183 positive flow (red arrows) based on upstream site HF183 detection and concentrations, as well as flow with no demonstrable HF183 detection (black arrows). Note that all maps presented were created and co-owned with the City of Airdrie and have been used with permission.

Table S1 Evidence of human sewage contamination and FIB results from investigative samples taken upstream of the N#1 site in Nose Creek during the first half of the investigation (weeks 1-3)

Sampling Date	Site	HF183	HumM2	*Enterococcus*	*E. coli*
		$\log_{10}$ copies/100 mL	$\log_{10}$ copies/100 mL	$\log_{10}$ CCE/100 mL	$\log_{10}$ MPN/100 mL
26/7/21	N1-15C2-N	4.06	DNQ	4.25	0.99
	N1-15C62-N	5.25	4.32	2.65	2.51
	N1-15C62-E	ND	ND	1.93	1.27
9/8/21	N1-10C39-N	ND	ND	2.33	0.80
	N1-10C43-E	ND	ND	ND	ND
	N1-10C43-W	ND	ND	1.71	0.30
	N1-15C54-N	ND	ND	3.79	0.61
	N1-15C17-E	ND	DNQ	4.57	1.78
24/8/21	N1-10C39-W	ND	ND	3.09	2.44
	N1-GenEdge-S	ND	ND	2.82	0.72
	N1-GenEdge-N	ND	ND	3.45	1.96
	N1-NoFrills-N	ND	ND	2.86	1.51

Table S2 Human sewage MST and FIB results from investigative samples taken upstream of the N#1 site in Nose Creek during the second half of the investigation (weeks 4-5)

Sampling Date	Site[a]	HF183	HumM2	*Enterococcus*	*E. coli*
		$\log_{10}$ copies/100 mL	$\log_{10}$ copies/100 mL	$\log_{10}$ CCE/100 mL	$\log_{10}$ MPN/100 mL
7/9/21	N#1	3.64	ND	2.19	0.98
	N1-10C39-E	ND	ND	2.35	ND
	N1-10C28-E	ND	ND	4.18	3.24
	N1-10C49-S	4.75	DNQ	2.32	1.71
	N1-10C15-E	ND	ND	1.43	0[b]
8/9/21	N1-15C62-N	4.40	ND	2.23	1.30
	N1-15C62-E	ND	ND	1.90	0.80
	N1-15C2-E	ND	ND	1.93	0[b]
	N1-15C2-S	ND	ND	2.57	0.93
	N1-15C17-W	ND	ND	2.67	0.99
	N1-15C20-W	DNQ	ND	2.57	1.38
	N1-16C104-E	ND	ND	1.84	0.61
	N1-16C104-N	ND	ND	2.45	0.72
	N1-16C106-E	ND	ND	2.12	1.13
27/9/21	N1-10C49-S	5.36	4.31	4.36	>3.38
	N1-10C41-N	6.08	6.05	5.60	>3.38
	N1-10C41-W	ND	ND	3.20	ND

N1-10C43-N	ND	ND	1.72	0.88
N1-10C39-E	ND	ND	2.81	0[b]

[a] Letters at the end of each manhole site name (i.e., N, E, S, or W) represent the direction of the specific trunk sampled at said manhole

[b] Due to $\log_{10}$ transformation, values of 0 represent detection at 1.00 MPN/100 mL for *E. coli*, and are not equivalent to an absence of detection at <1.00 MPN/100 mL.

Table S3 Conditions for all qPCR assays using the ABI 7500, including target, primer, and probe names, sequences, and concentrations where applicable

Target Name	Target	Target Organism (locus)	Primer/Probe	Primer/Probe Target Sequnce (5'-3')	Primer/Probe Conc.	Reference
**Fecal Indicator Bacteria (FIB)**						
Entero1	*Enterococcus* spp.	*23S* rRNA	Entero1-F	GAGAAATTCCAAACGAACTTG	1 uM	1, 2
			Entero1-R	CAGTGCTCTACCTCCATCATT	1 uM	
			Entero1-P	6FAM-TGGTTCTCTCCGAAATAGCTTTAGGGCTA-TAMRA	80 nM	
**Microbial Source Tracking (MST)**						
HF183	Humans	*Bacterioides* spp. (*16S* rRNA)	HF183-F	ATCATGAGTTCACATGTCCG	1 uM	3
			BFDrev	CGTAGGAGTTTGGACCGTGT	1 uM	
			BFD-FAM	FAM-CTGAGAGGAAGGTCCCCCACATTGGA-TAMRA	80 nM	
HumM2	Humans	Unknown (alpha-1,2-mannosidase analog)	HumM2-F	CGTCAGGTTTGTTTCGGTATTG	200 nM	4
			HumM2-R	TCATCACGTAACTTATTTATATGCATTAGC	200 nM	
			HumM2-P	FAM-TATCGAAAATCTCACGGATTAACTCTTGTGTACGC-TAMRA	125 nM	
**Pathogen Markers**						
VD16S	*Campylobacter* spp.	*16S* rRNA	VD16S-F	CTGCTTAACACAAGTTGAGTAGG	300 nM	5
			VD16S-R	TTCCTTAGGTACCGTCAGAA	300 nM	
			VD16S-P	6FAM-CGCTCCGAAAAGTGTCATCCTCC-BHQ1	100 nM	
invA	*Salmonella* spp.	*invA*	invA-F	GCGTTCTGAACCTTTGGTAATAA	450 nM	6
			invA-R	CGTTCGGGCAATTCGTTA	450 nM	
			invA-P	6FAM-TGGCGGTGGGTTTTGTTGTCTTCT-MGBNFQ	125 nM	
hsp60	*A. butzleri*	*hsp60*	Abutz-F	CTCTTCATTAAAAGAGATGTTACCAATTTT	300 nM	7
			Abutz-R	CACCATCTACATCTTC**W**GCAATAATTACT	300 nM	
			Abutz-P	6FAM-CTTCCTGATTGATTTACTGATT-NFQMGB	100 nM	
Stx1	STEC	*Stx1*	Stx1-F	CATCGCGAGTTGCCAGAAT	450 nM	8
			Stx1-R	GCGTAATCCCACGGACTCTTC	450 nM	
			Stx1-P	6FAM-CTGCCGGACACATAGAAGGAAACTCATCA-TAMRA	125 nM	
Stx2	STEC	*Stx2*	Stx2-F	CCGGAATGCAAATCAGTC	450 nM	8
			Stx2-R	CAGTGACAAAACGCAGAACT	450 nM	
			Stx2-P	6FAM-ACTGAACTCCATTAACGCCAGATATGA-TAMRA	125 nM	
**Sample Processing and Inhibition Controls**						

Sketa	*Onchorhynchus keta*	rRNA ITS	Sketa-F	GGTTTCCGCAGCTGGG	1 µM	2, 9
			Sketa-R	CCGAGCCGTCCTGGTC	1 µM	
			Sketa-P	VIC-AGTCGCAGGCGGCCACCGT-TAMRA	80 nM	

References:

1. Ludwig W, Schleifer KH. 2000. How quantitative is quantitative PCR with respect to cell counts? Syst Appl Microbiol 23:556–562. https://doi.org/10.1016/S0723-2020(00)80030-2

2. United States Environmental Protection Agency (US EPA). 2012. Method 1611: Enterococci in water by TaqMan® quantitative polymerase chain reaction (qPCR) Assay (EPA-821-R-12-008). https://www.epa.gov/sites/default/files/2015-08/documents/method_1611_2012.pdf

3. Haugland RA, Varma M, Sivaganesan M, Kelty C, Peed L, Shanks OC. 2010. Evaluation of genetic markers from the 16S rRNA gene V2 region for use in quantitative detection of selected *Bacteroidales* species and human fecal waste by qPCR. Syst Appl Microbiol 33:348–357. https://doi.org/10.1016/j.syapm.2010.06.001

4. Shanks OC, Kelty CA, Sivaganesan M, Varma M, Haugland RA. 2009. Quantitative PCR for genetic markers of human fecal pollution. Appl Environ Microbiol 75:5507–5513. https://doi.org/10.1128/AEM.00305-09

5. Van Dyke MI, Morton VK, McLellan NL, Huck PM. 2010. The occurrence of *Campylobacter* in river water and waterfowl within a watershed in southern Ontario, Canada. J Appl Microbiol 109:1053–1066. https://doi.org/10.1111/j.1365-2672.2010.04730.x

6. Daum LT, Barnes WJ, McAvin JC, Neidert MS, Cooper LA, Huff WB, Gaul L, Riggins WS, Morris S, Salmen A, Lohman KL. 2002. Real-time PCR detection of *Salmonella* in suspect foods from a gastroenteritis outbreak in Kerr County, Texas. J Clin Microbiol 40:3050–3052. https://doi.org/10.1128/JCM.40.8.3050-3052.2002

7. de Boer RF, Ott A, Güren P, van Zanten E, van Belkum A, Kooistra-Smid AMD. 2013. Detection of *Campylobacter* species and *Arcobacter butzleri* in stool samples by use of real-time multiplex PCR. J Clin Microbiol 51:253–259. https://doi.org/10.1128/JCM.01716-12

8. Chui L, Lee M-C, Allen R, Bryks A, Haines L, Boras V. 2013. Comparison between ImmunoCard STAT!® and real-time PCR as screening tools for both O157:H7 and non-O157 Shiga toxin-producing *Escherichia coli* in Southern Alberta, Canada. Diagn Microbiol Infect Dis 77:8–13. https://doi.org/10.1016/j.diagmicrobio.2013.05.015

9. Haugland RA, Siefring SC, Wymer LJ, Brenner KP, Dufour AP. 2005. Comparison of *Enterococcus* measurements in freshwater at two recreational beaches by quantitative polymerase chain reaction and membrane filter culture analysis. Water Res 39:559–568. https://doi.org/10.1016/j.watres.2004.11.011
